# Collaborative Localization and Location Verification in WSNs

**DOI:** 10.3390/s150510631

**Published:** 2015-05-06

**Authors:** Chunyu Miao, Guoyong Dai, Kezhen Ying, Qingzhang Chen

**Affiliations:** 1 College of Computer Science and Technology, Zhejiang Normal University of Technology, Liuhe Road No. 288, Hangzhou 310023, China; E-Mails: cymiao@zjnu.cn (C.M.); daiguoyong@gmail.com (G.D.); yingkz@163.com (K.Y.); 2 XingZhi College, Zhejiang Normal University, Jinhua 321004, China

**Keywords:** cooperative localization, wireless sensor networks, virtual force model, location verification, reliable localization

## Abstract

Localization is one of the most important technologies in wireless sensor networks. A lightweight distributed node localization scheme is proposed by considering the limited computational capacity of WSNs. The proposed scheme introduces the virtual force model to determine the location by incremental refinement. Aiming at solving the drifting problem and malicious anchor problem, a location verification algorithm based on the virtual force mode is presented. In addition, an anchor promotion algorithm using the localization reliability model is proposed to re-locate the drifted nodes. Extended simulation experiments indicate that the localization algorithm has relatively high precision and the location verification algorithm has relatively high accuracy. The communication overhead of these algorithms is relative low, and the whole set of reliable localization methods is practical as well as comprehensive.

## 1. Introduction

Wireless sensor networks are an important part of Internet of Things (IoT). Using a large number of sensor nodes, they form a self-organizing multi-hop network through wireless communication, and can be deployed in certain areas needing monitoring, with the aim of cooperatively sensing, collecting and processing the information within the coverage area, and transmitting these observations to the observers [1]. With the development of information technology like microelectronics, the usage of wireless sensor networks has gradually expanded from the military to various other fields, such as industry, agriculture, medicine, transportation, and so on [2,3]. The location of the node is an important parameter for sensed data. In general, the number of sensor nodes is enormous, hence it is impractical to measure the location of each node in advance. Due to the high energy consumption and cost, it is prohibitive to equip each sensor node with global positioning system (GPS) and besides, GPS cannot be used in sheltered environment such as indoor scenarios. Therefore, we usually adopt some indirect method to evaluate the locations of sensor nodes.

According to whether some location-aware nodes called anchor nodes need to be deployed in the WSN, the WSN localization algorithms can be divided into two categories: anchor-based localization algorithms [4] and anchor-free localization algorithms [5]. Usually, the localization accuracy of the former is better than that of the latter, therefore anchor-based localization algorithms are often used in scenarios which need high localization accuracy. Furthermore, according to whether the localization process needs to measure the distance between nodes, the localization algorithms can be range-based localization algorithm [6] and range-free localization algorithm [7]. Generally, range-based localization algorithms complete the establishment of the location coordinates system by measuring the distances between nodes through their received signal strength indicator (RSSI) [8], time of arrival (TOA) [9] or angle of arrival (AOA) [10] *etc.*, Among them, RSSI-based localization algorithms are the most practical and applicable.

In traditional static wireless sensor networks, the use of anchor nodes with preset location is widespread to reduce the application cost. We assume that all nodes are stationary, so the preset location information of all anchor nodes is reliable, but in practice the nodes may move accidentally due to natural or man-made factors. They may also send erroneous information due to malfunctions. In a hostile environment, the anchors even may be captured and deliberately provide incorrect location references. All this will cause great localization errors, which will influence the quality of service (QoS) of the WSN [11]. Therefore, when designing a localization algorithm for a WSN, we should take localization verification and node re-location process into consideration.

In view of the situations mentioned above, we design a lightweight distributed node localization scheme and a location verification algorithm based on a virtual force model, to achieve reliable localization in a WSN. Besides, we verify the algorithm by extensive experiments to evaluate the performance of these proposed algorithms. The contributions of this paper can be summarized as follows: (1) it proposes a model-consistent distributed node localization scheme as well as a location verification algorithm; (2) it considers the reliability difference of localization references between normal nodes and anchor nodes; (3) distance offset observations of neighbor nodes for a certain node in the WSN is combined by a sophisticated method. As far as we know, it is the first time a virtual force model has been included in WSN location verification. This paper is organized as follows: Section 2 gives a brief introduction to related work; problem modeling is given in Section 3; Section 4 provides a detailed description and theoretical demonstration of the algorithm; Section 5 verifies both the availability and the efficiency of the proposed algorithm by experiments, followed by conclusions and the future work in Section 6. The main notation used in this paper is listed in Table 1.

**Table 1:** Notation table.

Notations	Meaning
*m*	The number of anchor nodes
*n*	The number of normal nodes
*C_anchor_*	Coordinate of anchor
*C_normal_*	Actual coordinate of normal node
*C_e_*	Estimated coordinate of normal node
*d_ij_*	Actual pairwise distance
$\delta_{ij}$	Ranging distance
${\hat{d}}_{ij}$	Calculated distance after localization
∆	Localization error
*A_d_*	Collection of unreliable anchors
ω_1_ and ω_2_	Threshold in the two location refinement process
${\vec{f}}_{ij}$ and ${{\vec{f}}^{\prime }}_{ij}$	Amount of virtual force in the two location refinement process
${\vec{F}}_{i}$	Resultant
α *_j_*	Distance weight
*w_j_*	Reference weight

## 2. Related Work

In WSNs, localization methods fall into two categories according to whether some scheme has been adopted to get more reliable localization results, that is, unreliable localization methods and reliable ones. At present, there are some works concentrating on reliable localization in WSNs, which can be divided into range-based reliable localization algorithms and the range-free reliable localization algorithms [12]. We focus on the former. The research on reliable WSN localization can also be divided into outlier tolerant schemes [13] and reliable anchor selection schemes [14]. The former are applicable in scenarios with small ranging disturbances. They mainly focus on mitigating the localization reference effects of unreliable anchors, but if there are large errors in the reference locations, the localization accuracy will be greatly degraded. Our research belongs to the latter, so we review the state of the art of this area below.

### 2.1. Location Verification

In [15], a point-to-point localization verification algorithm that can be applied to any kind of ranging-based algorithm is proposed, but it requires nodes with GPS as the verified nodes. He *et al.* presented a localization algorithm based on an abnormality elimination which can be applied to TOA ranging technology [16]. Our research is based on RSSI ranging technology. Beacon Movement Detection (BMD) proposed by Kuo *et al.* [17,18] is mainly used to identify the anchors whose location has been changed passively in the network. That is, constructing a BMD engine in the network to collect all the RSSI information, which can identify whether the location of anchors has changed within a certain tolerance range. Usually, this kind of centralized algorithm has heavy communication traffic and sink nodes or background computers with strong computing power are required as well, hence it is not suitable for large scale randomly deployed WSN networks. There are certain related works which verify the anchor location by adopting a hidden localization verification station [19], which is also a centralized algorithm. In [20,21] rigidity theory is introduced to exclude outliers to provide reliable localization results, however, rigidity theory requires high ranging accuracy and it is computationally intensive. Garg *et al.* [22] proposed an anchor exclusion method by excluding the nodes who provided the largest gradient in the localization process to improve the reliability of localization, but they don’t consider the reference effect of normal nodes, and the method is unsuitable for anchor sparse networks, and in addition, it is also computationally intensive. Reference [23] designs a reliable localization algorithm using a distributed reputation model, but the response time when the network changes is relatively long. According to mutual observing information between neighbor nodes, Wei *et al.* [24] formulated a probability model to fulfill location verification, which achieved relatively good results, but they didn’t discuss the subsequent moved-node re-localization process. Reference [25] uses a distributed neighbor node scoring mechanism for RSSI to identify any drifted anchors, but it cannot be used in the case with compromised anchors.

### 2.2. Location Calibration

After recognizing the unreliable nodes in the network, we should not use their location information as the localization reference, but this may result in a lack of available anchors. As we know, using localized normal nodes as anchors to assist other normal nodes fulfill localization is a general method to resolve the problem of insufficient reliable anchors [26]. The key point of these methods is the localization reliability description of normal nodes. Adopting a stable quadrilateral model from graph theory [27,28] we can create a localization reliability model based on the geometric distribution of neighboring nodes. Yang *et al.* [29] described the localization reliability based on a probability model. Sheu *et al.* [30] presented a distributed localization algorithm in mobile WSNs, where the whole localization area of a located node is provided to other nodes, so there is the idea of reliable localization too. The abovementioned researches provide references for using a reliability model to judge the reliability of node location. However, the reliability judgment of node location should be better integrated with a localization algorithm to reduce computational overhead.

### 2.3. Virtual Force Model

Zou *et al.* [31] were the first to achieve node autonomous deployment in WSNs by adopting a virtual force model (VF model). As the VF model has the features of intuitive and easy operation, a large number of node deployment algorithms based on the VF model have been proposed. The so-called virtual force field assumes there are inter-forces (attraction or repulsion) between nodes, nodes and obstacles, as well as nodes and the deployment regions, and then reach the deployment target through the virtual force equilibrium. At present, there are a few methods using the VF model to achieve localization in WSNs. Reference [32] presented a WSN localization algorithm using a VF model, but it did not consider the reference differences between anchors and ordinary nodes in the virtual force computation. Owing to the introduced cumulative errors, when the location of located nodes is re-input to the localization process, there may be certain localization errors. By using the VF model in the location verification process, not only the amount of distance mismatches observed by neighbors of a certain node but also the direction of these mismatches are considered, which makes the verification process more comprehensive and effective. As far as we know, we are the first ones to introduce a VF model for location verification.

## 3. Problem Modeling

### 3.1. Motivation

According to whether a node knows its location in advance or not, WSN nodes fall into two classes: normal nodes and anchor nodes. Anchor nodes know their location, and normal nodes estimate their location based on the location of anchors through some mathematical method. The anchor cannot get its location by GPS in some shielded environments, so in general, the location is pre-established manually. The localization accuracy of range-based methods is relatively high, and a distance measured via RSSI has no need for extra devices, so many works focus on RSSI-based ranging localization methods. We aim to solve the localization and location verification problems in a certain scenario where nodes may drift and anchors may be malicious. In addition, the set of algorithms themselves must be consistent and scalable.

*Definition 1—Node Drifting*: in some scenarios, there may be some nodes’ locations that were moved passively, for example nodes moved by animals, and we call this kind of movement node drifting.

*Definition 2—Malicious Nodes*: Due to hardware malfunctions or for other reasons, some anchors broadcast wrong location reference information. Furthermore, in hostile environments, some anchors may be compromised to deliberately give other nodes wrong location references. We call these anchors malicious anchors.

We call drifted anchors and malicious anchors unreliable anchors because these anchors can cause a significant localization error. Normal nodes can eliminate these effects by re-locating themselves periodically, but after drifting or being compromised, these anchors can produce large negative effects on normal nodes’ localization process due to the location inconsistency between the claimed location and the real location of anchors. For range-based localization processes, the more anchors there are, the higher the localization accuracy that can be obtained, so some cooperative localization methods are proposed [19,25], which promote normal nodes as anchors, but certain accumulated errors would be introduced in such methods. In addition, distributed algorithms are more suitable for WSNs than centralized algorithms. To sum up, in this work, we want to present algorithms with the following features:

(1) A lightweight distributed localization algorithm for WSNs.
(2) A location verification algorithm which can detect drifted nodes and unreliable anchors.
(3) A re-located algorithm which adapts to anchor sparse WSNs.

### 3.2. Problem Statement

Let us assume that the total node number of a 2D deployed WSN is *N*. There are *m* anchors, denoted as *A* = {a*_i_*: *i* = 1, …, *m*} and *n* normal nodes denoted as *S* = {s*_i_*: *i* = 1, …, *n*}, where *m* + *n* = *N* and *m* << *n*. The coordinates of the anchors are *C_anchor_* = {*c_i_*: *i* = 1, …, *m*}, *c_i_* = [*x_i_*, *y_i_*]. The coordinates of the normal nodes are unknown, and we assume their coordinates are *C_normal_* = {*c_i_*: *i* = 1, …, *n*}, *c_i_* = [*x_i_*, *y_i_*]. The pairwise distance of neighbor nodes is $\delta_{ij}$ which can be acquired by Equation (1) in an ideal environment without noise-like errors is [4]:

(1) $$\delta_{ij}=10^{\frac{Rssi-E}{10n}}$$

where δ denotes the pairwise distance, *E* and *n* are constants which are relevant to the antenna gain and environment. Using the measured distance, node *n_i_* estimates its coordinates *C_e_* = [*x_e_*, *y_e_*] via a certain algorithm *f*(**·**). The real pairwise distance is *d_ij_* = ||*c_i_* −*c_j_*||; *i*, *j* = 1, …, *N*; Due to the measurement error, $\delta_{ij}$ = *d_ij_* + *d_ij_*·*noise_ij_*, *noise_ij_* ~ χ (0, σ^2^) (normal distribution, mean is 0, variance is σ^2^), results in *C_e_* ≠ *C_anchor_*. According to *C_e_* and *C_anchor_*, we get the localization distance of a certain node denoted as ${\hat{d}}_{ij}$, $\left|d_{ij}-{\hat{d}}_{ij}\right|=\Delta$, where ∆ is the localization error. 

All of nodes may have drifted or been compromised after the whole network was deployed. Let us assume the proportion of drifted and compromised nodes is relatively small, that is, the number is *t*. These nodes denoted as *A_d_* = {a*_k_*: *k* = 1, …, *t*, *t* << *m*}. The coordinates of anchors in collection *A_d_* broadcast coordinates *C′_anchor_*, which is different to their real coordinates *C_anchor_*, *i.e.*, *C_anchor_* ≠*C′_anchor_*. A larger localization error ∆ will be introduced when the normal nodes re-estimate their coordinates using *C′_anchor_*. Therefore, the objectives should be as follows:

(1) With *C_anchor_*, $\delta_{ij}$ and *C_e_* estimated from *f*(**·**), to minimize the ∆, *i.e.*,
  $$Min\;\left|d_{ij}-{\hat{d}}_{ij}\right|$$
(2) Construct a function *g*(**·**), to make *A'_d_* approximate to *A_d_*, *i.e.*,
  $$Min\;\left|A_{d}-{A^{\prime }}_{d}\right|$$

The terms *f*(**·**) and *g*(**·**) should be distributed, and the computation mechanism, including the computation data, should be consistent to reduce the computation overhead.

## 4. Cooperative Localization and Location Verification

### 4.1. Assumptions

(1) All of the nodes in the network have the same communication radius, *i.e.*, r, and the sensing model is an ideal circle.
(2) (The pairwise ranging distances of (*n_i_*, *n_j_*) are unbiased, *i.e.*, $\delta_{ij}$=$\delta_{ji}$.
(3) There are not collusions between these malicious anchors.
(4) All of the nodes can be drifted, but only the anchor nodes might be compromised.
(5) The proportion of unreliable nodes including drifted nodes and malicious anchors is lower than 50%. Otherwise, we cannot recognize the unreliable nodes [33].

### 4.2. Cooperative Localization Algorithm 

**Figure 1:**
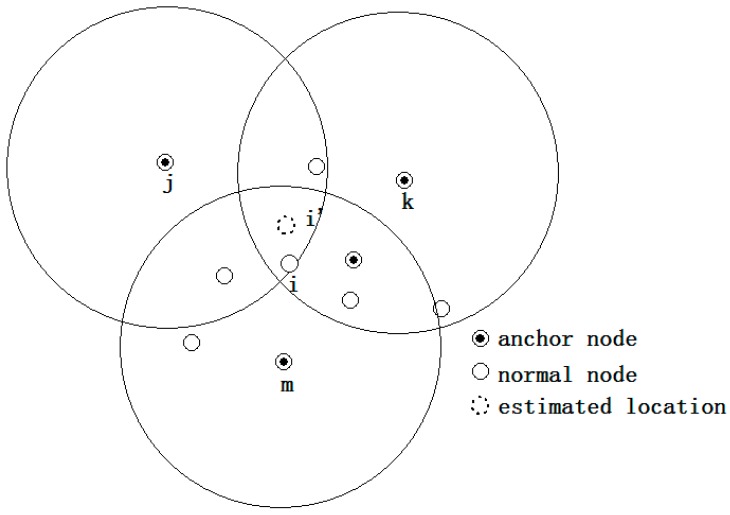
Initial location determination.

**Figure 2:**
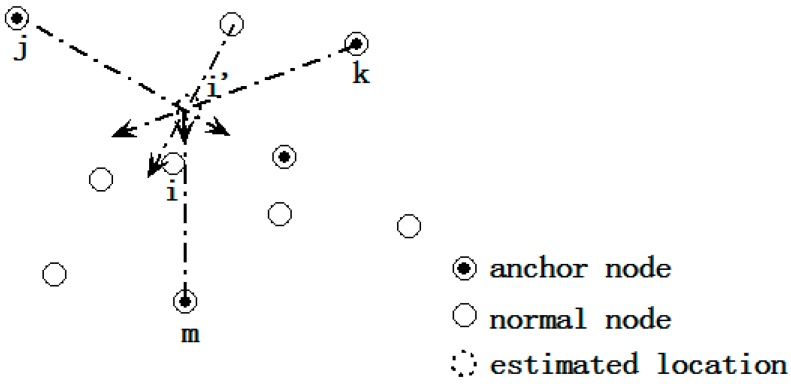
The force exerted on sensor.

From a relatively reliable initial location, sensor nodes move along the direction of resultant force to which these nodes are subject step by step. The step size is reduced when nodes move into a certain reasonable area. The iterative movements cease when the equilibrium of all force exerted on a certain node is reached. This is the main idea of the cooperative localization algorithm. We call this algorithm Virtual-force Localization Algorithm (VLA). In addition, as far as we know, this is the first time that node type and distance effect are considered when introducing the VF model in WSN node localization. There are three key steps in VLA:

(1) The initial location estimation: to enlarge the location search range, the three anchors whose ranging distances are the largest are selected to derive the initial location of a certain node that needs to be located. The centroid of the intersection of these three anchors is used as the initial location. As Figure 1 shows, *j*, *k* and *m* are the three farthest anchors of node *i*, and the initial location of node *i* is *i'*.
(2) Location adjustment: the initial location is adjusted to the final location using the virtual force model. As illustrated in Figure 2, the location of node *i* “moves” toward the correct position under the effect of virtual force that caused by other nodes. The movement ceases when the magnitude of the resultant force imposed on *i* is lower than the pre-set value ω_1_.
(3) Localization refinement: the step size of node “movement” is reduced so as to improve the localization accuracy. This iteration process ceases when the magnitude of the resultant force is lower than another pre-set value ω_2_ or when the iteration number reaches the pre-set *T*.

Several pairwise RSSI values are collected and are filtered by the Dixon guidelines [34] so as to overcome the effects caused by outliers. After filtering, the average of these RSSI values is used to estimate the pairwise distance. There are several virtual forces exerted on each node caused by other nodes in the VF model. The force between a pair of nodes is attractive when $\delta_{ij}$ > ${\hat{d}}_{ij}$, and the force expresses as a repulsion force when $\delta_{ij}$ < ${\hat{d}}_{ij}$, otherwise the force is zero. Each node updates its coordinates according to the direction of the resultant force by a fixed step size.

To achieve convergence rapidly, the magnitude of the virtual force in step (2) expressed as Equation (2) is larger than step (3) expressed as Equation (3). The force between a pair of nodes that cannot communicate with each other before drifting was expressed as a repulsion, that is,$\left(r-\delta_{ij}\right){\vec{e}}_{ij}$, where ${\vec{e}}_{ij}$ stands for the unit vector indicating the direction of the force and *r* is the communication radius of the sensor node:

(2) $${\vec{f}}_{ij}=\left({\hat{d}}_{ij}-\delta_{ij}\right){\vec{e}}_{ij}$$

The rationale behind Equation (2) is that the amount of the force between a pair of nodes is in direct proportion to the degree of mismatch of the ranging distance and the calculated distance according to the present localization iteration:

(3) $${{\vec{f}}^{\prime }}_{ij}=\left(1-\frac{{\hat{d}}_{ij}}{\delta_{ij}}\right){\vec{e}}_{ij}$$

The resultant force was expressed by Equation (4). This formula combines the neighbors’ observation of a certain node comprehensively, because not only the distance mismatches but also the direction of these mismatches are taken into account:

(4) $${\vec{F}}_{i}=\sum_{j=1}^{N}w_{j}\alpha_{j}{\vec{f}}_{ij}$$

In Equation (4) $\alpha_{j}=1-\frac{{\hat{d}}_{ij}}{D_{i}}$, denotes the distance weight. The ranging error is small when the interval between two nodes is small [4], so the value of this weight is inversely proportional to the interval. $D_{i}=\sum_{j=1}^{N}{\hat{d}}_{ij}$represents the sum of the distances of all anchors to a certain node that needs to be located. *w_j_* denotes the reference weight, that is, an anchor node has larger weight. The weight can be calculated by Equation (5):

(5) $$w_{j}=\left\{ \begin{array}{ll} 1, & if\;node\;j\;is\;an\;anchor\;node\\ {\left(0.9\right)}^{t}, & if\;node\;j\;is\;a\;normal\;node \end{array}\right.$$

where *t* stands for iterations. In order to reduce the accumulating error introduced by normal nodes, the weight of normal nodes decreases along with iterations. Thus far, we get the coordinate updating function of the node *i* with Equation (6):

(6) $$C_{ei}^{\left(t+1\right)}=C_{ei}^{\left(t\right)}+s{\vec{F}}_{i}$$

where *s* is the step size, and its value is a fixed percent of communication radius. The value of ω_1_ and ω_2_ are illustrated in Section 5. The pseudo code of the VLA algorithm is described in Algorithm 1.

**Algorithm 1** Location RefinementInput: ${\hat{d}}_{ij}$ and $\delta_{ij}$Output: Location of node *i*While (T > 0 and $\vec{F}$ < ω_2_)Location initiation; //centroid algorithmWhile ($\vec{F}$ < ω_1_) node *i* updates its location according to step size calculated by ${\vec{f}}_{ij}$;node *i* updates its location according to step size calculated by ${\vec{f}}_{ij}^{\prime }$;end;

### 4.3. Location Verification

There is an important difference between drifted nodes and malicious nodes, because the former participate positively in the drifted node recognition, while the latter don’t, so drifting can be detected according to the node’s observation of itself, while malicious anchor recognition merely relies on the mutual observation of all the neighbor nodes around a certain anchor.

**Figure 3:**
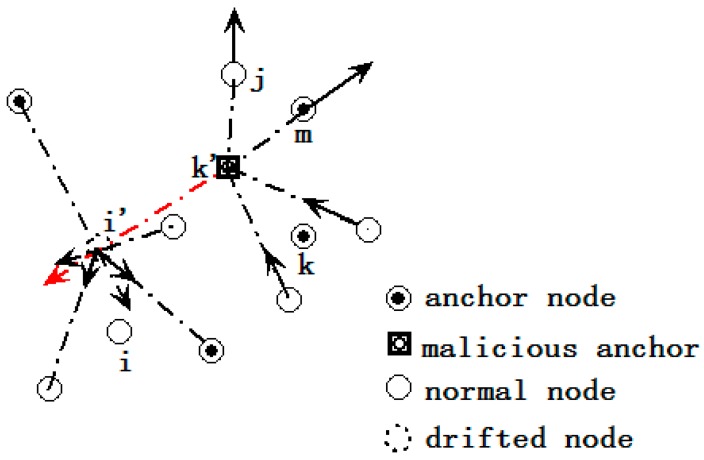
Drifted node and malicious node detection.

*Case 1 (Drifting recognition)*: Assuming node *i* is a drifted node (as Figure 3 shows). The δ*_ij_* is changed (denoted as δ'*_ij_*), while *d_ij_* still maintains its original value. The direction of the force exerted on *i* caused by its neighbors according to δ*'_ij_* points to *i*’s original location. Node *i* is regarded as a drifted node if the magnitude of the resultant force caused by its neighbors is larger than the threshold ω_3_, *i.e*., $\left|{\vec{F}}_{i}-{\vec{F}}_{i}^{\prime }\right|>\omega_{3}{\vec{e}}_{ij}$. It should be noted that node *i* cannot receive anchor *k*’*s* location reference broadcast, so there is no force caused on each other (line in red in Figure 3). Once a node identifies itself as a drifted node, then it broadcasts its declaration to its direct neighbors. The nodes who receive this declaration of a certain node remove the force caused by this node, and calculate the result again. Every node only responds to the declarations that come from other nodes once.

**Figure 4:**
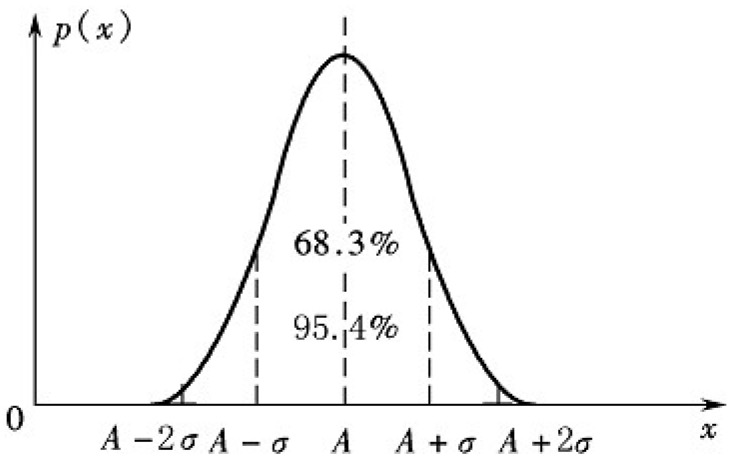
Probability distribution of RSSI.

The value of ω_3_ should ensure a lower false detection ratio in the case of there are some measurement noises in the ranging, whereas a higher success recognition ratio should be achieved. According to the object mentioned above, we analyze the RSSI noises by assuming the channel is an ideal Gaussian white noise channel. RSSI follows a normal distribution in that the mean is a real value and the standard deviation is σ. *i.e.*, ($P\sim N(P_{0}-10n_{p}\mathrm{lg}(d/d_{0}),\sigma^{2})$), as Figure 4 shows.

We set the value of ω_3_ as 2σ, and then the confidence interval is 95.4%. This means the RSSI difference will not exceed the threshold if there is no drift. For different networks, the value of σ should be determined by actual measurement of the RSSI in various environments.

*Case 2 (malicious anchor recognition)*: The pairwise forces between two nodes are interaction forces with opposite direction. Each node broadcasts the force caused by its neighbor anchors to 2-hop neighbors so each node can calculate the resultant force exerted on a certain anchor. The node recognizes a certain anchor as a malicious anchor if the magnitude of the resultant force is larger than the threshold mentioned above. These nodes omit the location reference of the certain anchor which has been regarded as malicious. We call this algorithm Virtual-force Location Verification Algorithm (VLVA). The pseudo code of VLVA is provided in Algorithm 2 below.

**Algorithm 2** Location VerificationInput: ${\hat{d}}_{ij}$and ${\delta^{\prime }}_{ij}$Output: ${A^{\prime }}_{d}$For each nodeif ($\left|{\vec{F}}_{i}-{\vec{F}}_{i}^{\prime }\right|>\omega_{3}{\vec{e}}_{ij}$) send message to its 2-hop neighbor nodes;recalculate ${\vec{F}}_{i}^{\prime }$ after removing the declared drifted node from its neighbor table;recognize drifted nodes and unreliable anchors;end

### 4.4. Re-Localization Algorithm

**Figure 5:**
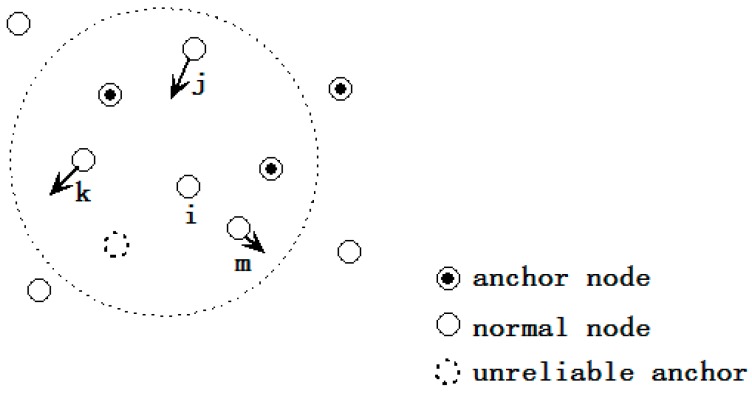
Temporal anchor selection.

A node uses the localization algorithm mentioned in Section 4.2 to re-locate itself when it is aware of having drifted. However, due to the fact that these unreliable anchors have been ignored, maybe the number of anchors around the drifted node is less than three, and this results in localization failure. Some located normal nodes can be promoted as temporal anchors to help other nodes fulfill their localization process. Aiming at achieving a high accuracy localization, the located nodes’ localization reliability must be taken into account. The magnitude of the residual resultant force left after the localization process can represent the localization reference reliability. It is important that the nearest node should be selected as temporal anchor if several nodes have same resultant force residual. The localization reference reliability of normal nodes can be expressed as follows:

(7) $$W_{r}=\alpha d_{ij}+\beta {\vec{e}}_{jR}$$

where *W_r_* stands for the weight of the reference reliability and *d_ij_* denotes the distance between node *i* and node *j*, and ${\vec{e}}_{jR}$ is the residual resultant force of node *j*, α and β are coefficients. Owing to the complicated relationship between ranging distance and localization accuracy, the values of α and β are determined by ground truth data matching in the experiment of Section 5. According to the value calculated by Formula (7), the node with a higher value has lower reliability. As shown in Figure 5, the drifted node *i* only has two real anchors in its communication range, so it selects the node *m* as temporal anchor due to its lower calculated value of *M*. The pseudo code of this process is provided in Algorithm 3.

**Algorithm 3** Re-localizationInput: ${A^{\prime }}_{d}$ and ${\delta^{\prime }}_{ij}$Output: location of each nodeFor each drifted nodePseudo anchor selection;call Algorithm 1;end

### 4.5. Analysis of Effectiveness and Complexity

The location service of WSN will not be degraded significantly as long as the average localization error is lower than 40% [26]. Let us assume the value of σ is lower than 10% of communication radius, we get:

**Theorem 1.** *The distributed VF model based localization algorithm can converge effectively*. 

**Proof of Theorem 1.** All of normal nodes can complete step (1) due to the fact that anchor nodes know their own coordinates. The difference between step (2) and step (3) is the magnitude of the virtual force. The location variation in a certain round only affects the next iteration. The tiny vibration of location in these iterations will cease when the iteration termination threshold was reached.

**Theorem 2.** *The drifted nodes and malicious anchors should be detected with a high probability in case of the number of these nodes and anchors is no more than 50% of the total number of nodes*. 

**Proof of Theorem 2.** The location of each node is determined by the effect of the resultant force caused by its neighbors. The distribution of drifted nodes is evenly distributed, and there is no collusion between malicious nodes. The majority of nodes provide correct localization references, thus, the magnitude of resultant force of a certain node is reduced or enlarged by these unreliable nodes. We assume the node distribution density of an unreliable node is ρ, and the node distribution is a Poisson distribution, then the probability that the number of unreliable nodes in a certain node’s communication range reached *k* can be derived from Formula (8):

(8) $$P(N=k)=\sum_{i=0}^{k}\frac{{\left(\rho \times A\right)}^{i}}{i!}e^{-(\rho \times A)}$$

where *A* is the communication area of a certain node, so the probability that a large number of unreliable nodes is gathered in a certain area is pretty small. Furthermore, even if there are 50% unreliable nodes around a certain node, due to the fact the ranging error is distributed evenly and the location inconsistency is random, the probability of these unreliable node forming an identical resultant direction which be opposite to the original direction is ${\left(\frac{1}{360}\right)}^{k}$.

To sum up, the recognition success probability of the proposed method is very high.

*The time complexity of these algorithms*: Let us assume the localization iterations is *n*, Each node calculates the force value *k* times per iteration, and *k* is the network connectivity with a pretty low value, so the time complexity is *O*(*n*).

*Communication overhead*: The maximum communication overhead is used to detect malicious anchors. Every node broadcasts the force exerted on some certain anchors in the 2-hops local area, so the communication overhead is relevant to the average network connectivity. We assume the average network connectivity is *k*, then the communication overhead of each node is *O*(*k*). In addition, the ranging result in drifted nodes and malicious detection can be used as input of the re-locate process, so, the whole set of algorithms has consistency, and hence the whole set of algorithms is comprehensive.

## 5. Simulations and Discussion

Simulations are conducted in a square with 500 m × 500 m area. There are *n* normal nodes and *m* anchors that are deployed randomly. The interval of each pair of anchors is more than 5 m. The communication radius of each node is 100 m. Ranging error with [−10% 10%] of actual pairwise distance was introduced in each distance estimation. After the deployment and the first localization, there are *t* anchors changed to unreliable anchors, their location changes are more than 20 m, and among them, the proportion of compromised anchors is less than 45%. Each experiment is conducted 50 times.

### 5.1. Localization Accuracy

The ratio of Root Mean Square (*RMS*) to the communication radius *R* is adopted as an indicator to evaluate the localization accuracy. We call this ratio Localization Error Rate (LER), and the RMS is defined as follows:

(9) $$RMS=\sqrt{\frac{1}{n}{\sum \left\|d_{ij}-{\hat{d}}_{ij}\right\|}^{2}}$$

In order to validate the localization accuracy and the robustness of VLA, we divide the experiment into two parts: (1) value of *n* is fixed at 200, and *m* varies from 10 to 40 with step size 5; (2) the number of anchors is fixed at 20, and the number of total nodes varies from 100 to 400 with step size 50. The former verifies the dependency of localization accuracy on anchor density, the later tests the performance of our algorithm in various network connectivity scenarios. The value of ω_1_ is 30% of the communication radius, and ω_2_ is 2σ.

**Figure 6:**
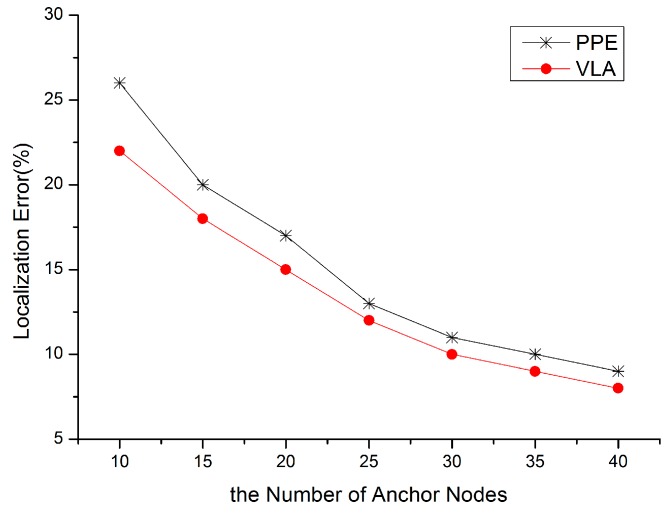
Anchor density *vs.* localization error.

Reference [32] also adopted a distributed virtual force model to fulfull localization, so we evaluate the performance of our VLA by comparing with PPE in [32]. As shown in Figure 6. the localization error of LVA is lower than PPE for various anchor densities. The reason is that VLA takes the two weights (*i.e.*, the localization reference weight and the distance wieht of anchor) into account. 

**Figure 7:**
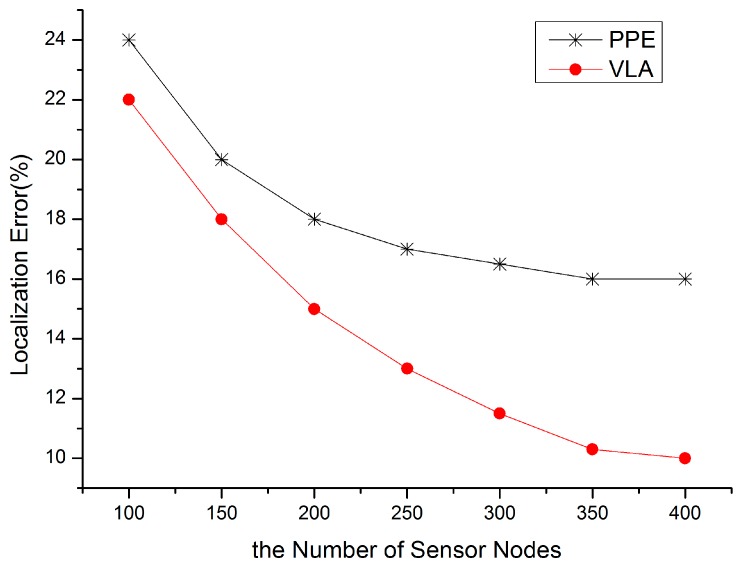
Node density *vs.* localzation error.

Figure 7 shows that VLA has a lower localization error in a dense network, also for the same reason. Whether in a dense network or in a sparse network, the localization performance of VLA is relatively good. Although the location error is on the order of 10%–20% in most scenarios due to the introduced ranging errors, the location accuracy is more than 60%, so the proposed algorithm is usable in most cases.

### 5.2. Performance of Location Verification

The recognition success rate and the recognition error rate are selected to evaluate the performance of our VLVA. The former is defined as $\left|A_{d}\cap {A^{\prime }}_{d}\right|/\left|A_{d}\right|$, and the latter is defined as $\left|A_{d}-{A^{\prime }}_{d}\right|/\left|A_{d}\right|$. The recognition success rate is a ratio of the number of nodes considered as unreliable to the number of actual unreliable nodes. The recognition error rate is a ratio of the number of nodes considered as unreliable nodes by erroneous judgment to the number of actual unreliable nodes. The DBDD algorithm in [25] is used to compare with our VLVA. The number of drifted anchors is fixed, and the density of network is varied from 100 to 400. 

**Figure 8:**
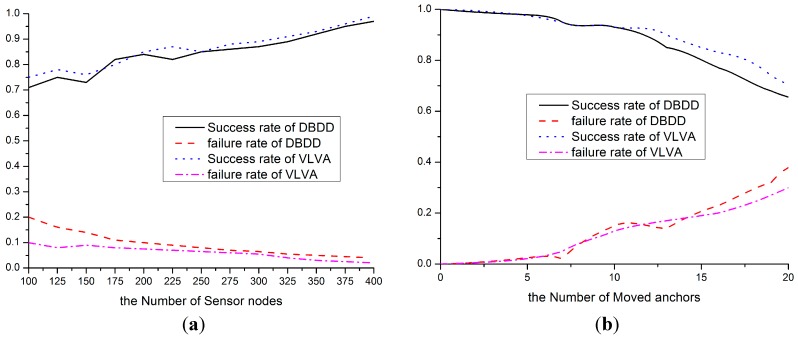
Location verification performance. (**a**) Performance vs number of anchor node; (**b**) Performance vs number of moved anchor node.

The success rates of these two algorithms increases as the node density increases, and the error rates of the two decline correspondingly. The reason is that the quantity of reference nodes is increased, but the recognition performance of VLVA is better than DBDD as shown in Figure 8a, because VLVA takes not only the changing scope of RSSI but also the movement direction of node into account. Figure 8b shows that both algorithms have relatively low success rate and relatively high error rate in the circumstance where the quantity of unreliable anchors is large. However, the decline of performance of VLVA is relatively gentle. The reason also is the movement direction being considered so that the cooperative judgment of neighbor nodes can be made reliable.

### 5.3. Re-Localization Performance 

**Figure 9:**
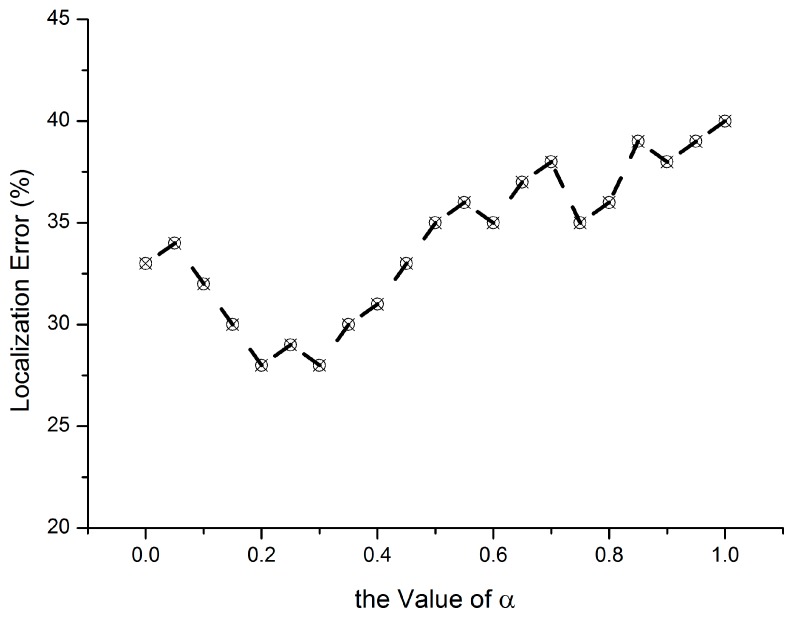
Coefficients’ variation *vs.* localization error.

In the process of node re-localization, the values of α and β directly affect the result of temporal anchor selection. Matching with the real measurement data, a temporal anchor selection experiment is conducted, as shown in Figure 9, where α + β =1. The temporal anchor can provide good reference performance when the value of α is between 0.2 and 0.3. To verify the necessity of temporal anchor selection, we design another algorithm called A1 which randomly selects a node as anchor. 

**Figure 10:**
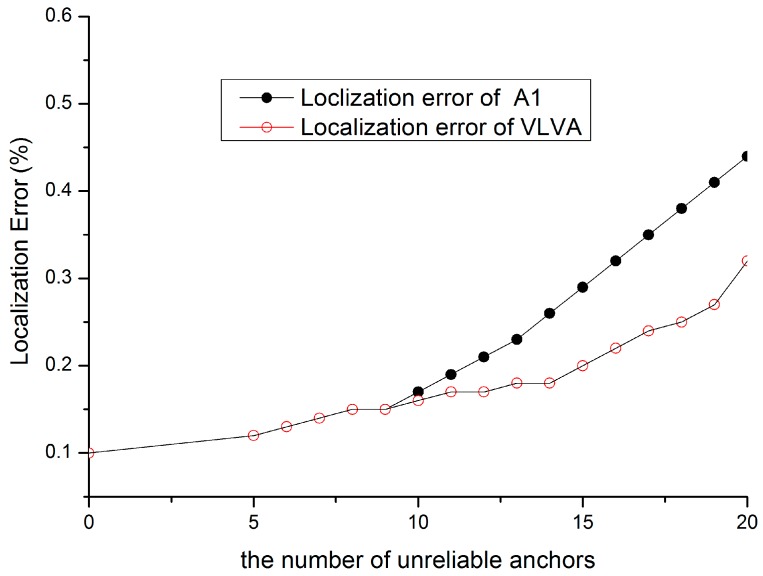
Performance of re-localization.

Figure 10 shows the comparison result between A1 and our VLVA. As we can see, the localization error of VLVA is low due to the anchor selection scheme. Because there is no other anchor which can provide reliable localization reference in the re-localization, it is necessary to enlarge the weight of nodes with high reliability by temporal anchor selection.

### 5.4. Communication Overhead

**Figure 11:**
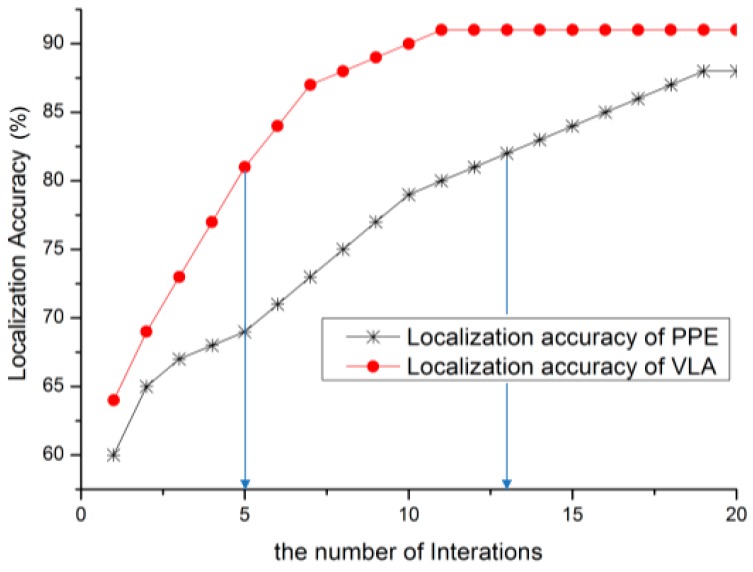
Comparison of communication overhead.

The proposed algorithm is distributed, so the communication overhead increases linearly along with the increasing network size. The algorithm in [32] also is a distributed one, and used here to compare with our VLA. The experiment result shows (see Figure 11) the VLA converges rapidly compared to the algorithm in [32] under the same circumstances. 

For example, the PPE algorithm takes 13 rounds of iteration to reach 80% average localization accuracy, whereas VLA only takes 5 rounds. Due to the use of the same communication model, the less iteration there is, the less communication overhead there is. The proposed VLA uses the centroid of the intersection of anchors as the initial location which is better than PPE that uses the average coordinates as initial location. In addition, normal nodes were allocated a low weight, so the location vibration is reduced in the localization process.

## 6. Conclusions

In view of some certain scenarios with drifting nodes and unreliable anchors, a set of localization and location verification algorithms is presented. The virtual force model is introduced to represent the relative localization error. A cooperative observation method is used to detect these unreliable nodes in WSNs, and a temporal anchor selection scheme is adopted to re-locate the drifting nodes. Extensive experiments show that these algorithms are both practicable and effective. In future work, some field experiments should be conducted to verify the performance of these proposed algorithms, besides of this, the verification of the moving anchor path in mobile WSNs is also a prospective issue.
